# Quantification of COVID-19 Vaccine Needle and Syringe Dead Space Volumes

**DOI:** 10.7759/cureus.18969

**Published:** 2021-10-22

**Authors:** Derek M Smith, Samuel L Weiss, Kevin M White

**Affiliations:** 1 Allergy/Immunology, San Antonio Uniformed Services Health Education Consortium, San Antonio, USA

**Keywords:** vaccine safety, vaccine wastage, vaccination process, dead space volume, quality improvement, covid-19 vaccine

## Abstract

Introduction: The COVID-19 pandemic taught many lessons regarding vaccine manufacturing, transportation, and delivery. Throughout the vaccination campaign, different vaccination sites reported that six or seven doses of the Pfizer/BioNTech COVID-19 vaccine could be reliably withdrawn from each multidose vial. This discrepancy was hypothesized to be caused by the differences in needle and syringe dead space volumes with differing supplies utilized at each site, but independent data regarding these volumes are lacking; therefore, we sought to objectively evaluate the volume of dead space for supplies commonly used for immunizations.

Materials and Methods: Available needles and syringes of different brands and specifications that could be used for administering vaccines were gathered. Each needle and/or syringe was weighed with a scientific, calibrated scale upon removal from the manufacturer's packaging and then filled with distilled water to simulate standard vaccine preparation. The water was then expelled, simulating vaccination, and then reweighed on the same scientific scale. The difference between the two weights was secondary to the water remaining within the needle or syringe after the simulated use, which was equivalent to the dead space volume.

Results: Manufacturer design, gauge, needle length, and syringe volume separately correlate with volume differences. Intuitively, the needles and syringes with the most dead space were consistently the 1.5-inch needles and the larger volume syringes, regardless of the manufacturer.

Conclusion: The withdrawal of individual vaccine doses from a multidose vial inevitably results in vaccine volume loss due to the dead space of needles and syringes. As such, reliably obtaining seven doses of the Pfizer/BioNTech COVID-19 vaccine is improbable without utilizing specialized low dead space supplies.

## Introduction

Safe and effective COVID-19 vaccines were developed in record time, but vaccine delivery has been accompanied by procedural nuances and logistical complexities. The Pfizer/BioNTech COVID-19 vaccine was the first COVID-19 vaccine approved by the US Food and Drug Administration (FDA) under an “Emergency Use Authorization” on December 11, 2020 [1]. Days afterward, vaccine shipments began arriving at hospitals nationwide for immediate utilization to vaccinate healthcare workers and emergency first responders. The vaccines are provided by the manufacturer in multidose vials that require dilution with a 0.9% sodium chloride solution before the withdrawal of individual doses. The manufacturer's instructions mandate that 1.8 mL of this diluent is added to the 0.45 mL vaccine concentrate supplied in the vial for a total volume of 2.25 mL after dilution [2]. As each Pfizer COVID-19 vaccine dose is 0.3 mL, it quickly became mathematically apparent that there might be remaining vaccine volume in the multidose vials after the five FDA-approved doses were withdrawn.

As vaccination began, theory became reality where six doses of vaccine were consistently obtained from each multidose vial. The FDA officially approved expanding the number of available doses in each vial to six or even seven doses, if possible, on December 23, 2020 [3]. Concurrently, the media began reporting that some locations were even able to consistently obtain seven 0.3 mL doses per vial using special equipment and techniques; however, this feat could not be reproduced at many vaccination locations. These assertions caused questions among vaccine experts as, while theoretically possible (2.1 mL for seven 0.3 mL doses subtracted from 2.25 mL of total vaccine volume equals 0.15 mL possible remaining volume), obtaining seven doses per vial might not account for the unavoidable volume loss caused by needle dead space when withdrawing individual vaccines from a multidose vial. Efforts to find academic research to quantify this volume were unsuccessful, and not all manufacturers comment on this volume on their product’s packaging. In response, we sought to attempt to independently quantify the amount of needle dead space in common ancillary vaccine supplies.

## Materials and methods

In order to accurately enumerate this dead space volume, we obtained as many different hypodermic needles and syringes as possible given the current limited supply conditions found during the COVID-19 pandemic. Each needle and/or syringe was weighed with a scientific, calibrated scale upon removal from the manufacturer's packaging and then filled with 0.5 mL distilled water to simulate standard vaccine preparation. The distilled water was then expelled completely into a waste container simulating vaccination of a patient. The needle and syringe were then reweighed using the same scale. The difference between these two weights was attributed to the weight of the water remaining in the needle dead space. As 1 g of water equates to 1 mL of water, these differences in weights were reported as equitable volumes. This process was repeated in quadruplicate to ensure data consistency. Means and standard deviations were then calculated.

## Results

The results of our investigation are reported in Table 1. These are the first data we are aware of when attempting to independently quantify the volume of dead space that can be expected from the syringe and needle combinations provided with the COVID-19 vaccines. Of note, Pfizer recommends using 22-25 gauge needles of 1-1.5 inches in length for administration. The one-inch needles with separate syringes confer approximately 0.05 mL of dead space per vaccine based on our measurements as shown below. 

**Table 1:** Dead space volumes of vaccination supplies

Vaccination Product	Specific Details	Mean Dead Space (mL)	Standard Deviation (mL)
Fixed needle and syringe combinations	VanishPoint® 25G x 1" with 1 mL syringe	0.0103	0.0049
	VanishPoint® 23G x 1" with 3 mL syringe	0.0750	0.0105
Syringes	BD® 1 mL syringe without needle	0.0343	0.0114
	BD® 3 mL syringe without needle	0.0179	0.0110
	DPS® 1 mL syringe without needle	0.0104	0.0029
Needles	Magellan® 23G x 1" needle without syringe	0.0309	0.0097
	Magellan® 25G x 1" needle without syringe	0.0478	0.0015
	SolCare® 25G x 1" needle without syringe	0.0652	0.0042
	DPS® 23G x 1.5" needle without syringe	0.0668	0.0070
	TKMD® 21G x 1.5" needle without syringe	0.0742	0.0013

## Discussion

It remains mathematically improbable to be able to dependably obtain seven 0.3 mL doses from a Pfizer multidose vial with the current standard vaccination supplies as shown above. In the effort to maximize the efficiency of the COVID-19 vaccination campaign, hospitals and mass vaccination sites have appropriately attempted to innovate their standard approach to vaccination. Utilizing low dead space needles and syringes is clearly helpful to improve efficiency by reliably obtaining a sixth dose of vaccine per multidose vial. However, some techniques are not recommended.

For example, using a single needle to withdraw the vaccines from the multidose vial into separate syringes and then using a new “dry” needle for each injection may allow for seven doses per vial. Unfortunately, this technique likely underdoses each patient by the volume of vaccine remaining in the new injection needle’s dead space. With a standard vaccine dose of 0.5 mL, this 0.05 mL dead space difference would cause the dose of the vaccine to be reduced by 10%. With the smaller 0.3 mL dose approved for the Pfizer COVID-19 vaccine, the dose could be reduced by 17% using a new, dry injection needle. While the clinical implication of a lower dose of vaccine is not established, it is not recommended by the Centers for Disease Control’s Advisory Committee on Immunization Practices. While these data were derived from federally supplied COVID-19 vaccine needles and syringes, they have broad applicability as these supplies are widely commercially available and will result in dead space losses irrespective of what they are used to inject.

## Conclusions

The withdrawal of individual vaccine doses from a multidose vial inevitably results in volume loss due to the dead space of needles and syringes. Now with unbiased data quantifying the volume of these losses, immunization programs can maximize vaccine preparation efficiency without inadvertently underdosing patients. Differing needle and syringe brands and item specifications can produce replicable results. These data will hopefully guide supply ordering preferences in order to maximize efficiency. The concept of dead space volume loss applies to other injectable vaccines and medications and should be accounted for when withdrawing these products from multidose vials to ensure correct dosing to patients.
